# Diagnostic Accuracy of Doppler Twinkling Artifact for Identifying Urinary Tract Calculi

**DOI:** 10.7759/cureus.5647

**Published:** 2019-09-13

**Authors:** Hatem Adel, Amjad Sattar, Anila Rahim, Anum Aftab, Syed Omair Adil

**Affiliations:** 1 Radiology, Dow University of Health Sciences, Karachi, PAK; 2 Epidemiology and Public Health, Dow University of Health Sciences, Karachi, PAK

**Keywords:** twinkling artifact, diagnostic accuracy, sensitivity, renal colic

## Abstract

Introduction

Flank pain is a frequent cause of emergency department visits and is often due to renal or ureteric colic. Ultrasound is often the initial imaging study used for the detection of urinary tract calculi. Twinkling artifact is a Doppler artifact usually seen on echogenic rough surfaces such as calculi. Its presence can improve the sensitivity and specificity of ultrasound in stone detection. The objective of the current study was to determine the diagnostic accuracy of the Doppler twinkling artifact for detecting urinary calculi using non-contrast computed tomography as the gold standard.

Materials and methods

In this cross-sectional study, both male and female patients of any age having flank pain, burning micturition with or without hematuria were included. Ultrasound was performed and the presence or absence of Doppler twinkling artifact on calculus was noted. Following ultrasound, patients underwent plain CT scan and findings of stones were documented. Sensitivity, specificity, positive and negative predictive values, and diagnostic accuracy of Doppler twinkling artifact was calculated considering CT findings as the gold standard.

Results

Out of the total 221 patients, 146 (66.1%) were males and 75 (33.9%) were females. The mean age of the patients was 45.98 ± 16.30 years. Urinary tract calculi on ultrasound were observed in 74 (33.50%) patients, while on CT urinary tract calculi were observed in 127 (57.50%) patients. Diagnostic accuracy of Doppler twinkling artifact was found to be 71.49% with sensitivity, specificity, positive predictive value, and negative predictive value of 54.33%, 94.68%, 93.24%, and 60.54%, respectively.

Conclusion

Doppler twinkling artifact has low sensitivity, high specificity, and suboptimal diagnostic accuracy for the diagnosis of urinary tract calculi. Integration of this artifact has a lower sensitivity as compared to non-contrast CT scan. Multicentric studies with larger sample size and focusing on interobserver and intraobserver variability are recommended to have a consensus regarding Doppler twinkling artifact in the evaluation of renal and ureteric calculi.

## Introduction

Flank pain is a common reason for presentation to the emergency department (ED). It is most commonly caused by urinary tract obstruction due to calculi. It has a variable prevalence worldwide [1]. Genetic and environmental factors are possible contributing factors to the development of stones [2]. Urolithiasis has a prevalence rate of 12% in Pakistani population [3].

Ultrasonography is a safe, inexpensive and non-invasive imaging tool that is widely available and has an acceptable accuracy in the evaluation of kidney and bladder anatomy. It has B-mode, i.e., the grayscale mode and color flow mode that is color Doppler mode. According to one study the sensitivity and specificity of ultrasound for diagnosing urinary tract calculi is 84% and 53% respectively [4]. It has an advantage of easy availability, low cost and no ionizing radiation.

In 1996 twinkling artifact was described by Rahmouni as an artifact generated by a strongly reflective medium on color Doppler ultrasonography [5]. Twinkling artifact appears as a rapidly alternating color Doppler signal behind certain strongly reflecting stationary irregular objects [6]. After the description of twinkling artifact, it has been reported mainly in association with urinary calculi [7,8]. It has sensitivity and positive predictive value of 83% and 94% respectively in diagnosing urinary tract calculi [9]. Another study showed the sensitivity, specificity, positive predictive value and negative predictive value of Doppler twinkling artifact in diagnosing urinary tract calculi to be 56%, 74%, 62%, and 68%, respectively [10].

With more recent advancements, there is a trend towards the increased use of non-contrast computed tomography (CT) scan for evaluation of patients with renal or ureteric colic. It is considered very effective for the detection of renal or ureteric calculi [11-12]. Low dose non-contrast CT has a sensitivity of 96.6% and specificity of 94.9% for urolithiasis [13]. However, there is exposure of the patient to the ionizing radiation. Thorough literature search yielded scarce data on this topic from a developing country. Therefore, the aim of this study was to determine the diagnostic accuracy of Doppler twinkling artifact for identifying urinary calculi using non-contrast CT as the gold standard.

## Materials and methods

This cross-sectional study was conducted at the Dow Institute of Radiology, Dow University of Health Sciences from 17th May 2016 to 17th November 2016. Both male and female patients of either age having complaints of flank pain with burning micturition for any duration were included. Patients were excluded if they had acute or chronic renal failure or urinary tract infection. Patients were also excluded if they were already diagnosed as having urinary tract calculi and presented for follow-up. Informed consent was taken from all patients before enrolling them in the study. Only those patients were included in the study that followed the inclusion and exclusion criteria. Patients were enrolled before undergoing a CT scan; therefore, while performing the sonographic examination, it was unknown whether there was a calculus present or not. CT scan was performed on the 16-slice CT scanner (BrightSpeed, GE Medical Systems, Milwaukee, WI, USA). A radiologist who was blinded to sonography results evaluated the CT scan for stone in kidneys, ureters, and urinary bladder. While the patient was waiting to undergo CT scan, the patient underwent a limited sonographic scan of kidneys, ureters, and urinary bladder. This examination was performed on ultrasound machine with color Doppler capability (Voluson S6, GE Medical Systems, Milwaukee, WI, USA) by a trained sonographer having three years of experience using a curved low-frequency ultrasound probe. The urinary tract was evaluated with both grayscale and color Doppler ultrasound and the sonographer was blinded to the CT scan results and described the location of calculus and the presence or absence of twinkling artifact on urinary calculi on color Doppler scan. Data was entered and analyzed using Statistical Package for Social Sciences (SPSS) version 22. Quantitative outcome variables such as age and duration of symptoms were mentioned as mean and standard deviation. Qualitative outcome variable such as gender was mentioned as frequency and percentage. Diagnostic accuracy including sensitivity, specificity, positive predictive value (PPV), and negative predictive value (NPV) of the twinkling artifact was calculated using contingency tables using CT scan findings as the gold standard.

## Results

In total, 221 patients were included in the study. The mean age of the patients was 45.98 ± 16.30 years. In total, 146 (66.10%) were males and 75 (33.9%) were females. The mean duration of symptoms was 12.62 ± 9.76 hours. Baseline characteristics of the patients are summarized in Table 1.

**Table 1:** General characteristics of the patients

General characteristics of the patients (n=221)		
	n	%
Age, years	45.98 ±16.30^ǂ^	
≤45 years	111	50.2
>45 years	110	49.8
Gender		
Male	146	66.1
Female	75	33.9
Duration of symptoms, hours	12.62 ±9.76^ǂ^	
^ǂ^mean±SD, n: number		

Urinary tract calculi on ultrasound were observed in 74 (33.50%) patients while on CT urinary tract calculi were observed in 127 (57.50%) patients (Table 2).

**Table 2:** Ultrasound and non-contrast CT results

Ultrasound and non-contrast CT results (n = 221)			
Ultrasound findings	Non-contrast CT findings		Total
	Positive	Negative	
Positive	69	5	74
Negative	58	89	147
Total	127	94	221

Sensitivity, specificity, positive predictive value, negative predictive value, and diagnostic accuracy of Doppler twinkling artifact was found to be 54.33%, 94.68%, 93.24%, 60.54%, and 71.49%, respectively.

## Discussion

Ureteric and renal colic are common causes of acute flank pain in patients presenting in emergency departments. Its exact incidence and prevalence varies with respect to age, gender and ethnicity [14]. For suspicion of any undiagnosed stone disease of urinary tract, non-contrast CT scan is commonly employed. It has a major disadvantage of utilizing ionizing radiation. The current study evaluated the performance of Doppler twinkling artifact in the diagnosis of stone disease of the urinary tract.

The current study showed that the performance of sonographic Doppler twinkling artifact was not optimum for the diagnosis of calculus when compared with reference standard non-enhanced CT scan. The sensitivity of twinkling artifact was found almost comparable to the one reported by Dillman et al. [15]. However, it was lower as compared to that reported by other authors [16-17]. The specificity of twinkling artifact was higher as compared to other studies [16,18]. Ultrasound is a highly operator-dependent modality and this dependency may be responsible for varying sensitivity and specificity of Doppler twinkling artifact for identification of stones on ultrasound. Another hypothesis for the low sensitivity of twinkling artifact could be a low pulse repetition frequency of the Doppler setting when evaluating the stone.

The false-positive rate of Doppler twinkling artifact was high. A similar finding has been reported by a previous study [9]. False negatives were also reported by our study. The etiologies of these false positives and false negatives could be related to different reasons such as stone density and its chemical composition, presence of echogenic fat in the renal sinus that sometimes may mimic a stone, setting of ultrasound machines, or even the generation of ultrasound machine [19]. False-positive diagnosis of urinary tract calculi can usually prompt for a CT scan that was not needed for evaluation. The possible factors of false positives could be renal artery calcification or highly echogenic areas within the renal sinus [5].

In the current study, the positive predictive value of sonographic twinkling was comparable to the one reported by the previous study [9]. Another study has reported a lower positive predictive value for reporting urinary tract calculi [15]. Sonographic Doppler twinkling is a subjective finding and may depend on the capability of a radiologist/sonographer along with the sonographic technique. The accuracy of Doppler twinkling artifact was also lower in our study as compared to another study [20].

Few limitations need consideration for the current study. One of them was that we only utilized a single ultrasound machine for the current study. Doppler Twinkling artifact is dependent upon the machine’s intrinsic settings and this could affect the diagnosis [19]. Another limitation of our study was that interobserver and intraobserver reliability was not evaluated.

Despite these limitations, we believe that this study was a step in the evaluation of utilization of Doppler twinkling artifact for diagnosis of stone disease of the urinary tract. The results of the current study provide local insights regarding the diagnostic accuracy of Doppler twinkling artifact. In addition, the results of current study suggest that this artifact, if integrated into routine ultrasound practice for the evaluation of ureteric or renal stones, has a lower sensitivity as compared to non-enhanced CT scan.

It is recommended that further studies in multiple centers, having a larger sample size and more variables such as patient’s performance status and clinical and laboratory parameters, should be undertaken. Moreover, interobserver and intraobserver variability should be calculated to have a consensus regarding the radiologist agreement on Doppler twinkling artifact for the detection of renal or ureteric stones.

## Conclusions

The findings of this study report a low sensitivity, high specificity, and suboptimal diagnostic accuracy of the Doppler twinkling artifact for the diagnosis of urinary tract calculi. Integration of this artifact has a lower sensitivity as compared to non-contrast CT scan. Multicentric studies with larger sample size and focusing on interobserver and intraobserver variability are recommended to have a consensus regarding Doppler twinkling artifact in the evaluation of renal and ureteric calculi.

## References

[1] Rodgers AL (2013). Race, ethnicity and urolithiasis: a critical review. Urolithiasis.

[2] Monico CG, Milliner DS (2012). Genetic determinants of urolithiasis. Nat Rev Nephrol.

[3] Rizvi S, Naqvi S, Hussain Z (2002). The management of stone disease. BJU Int.

[4] Fulgham PF, Assimos DG, Pearle MS, Preminger GM (2013). Clinical effectiveness protocols for imaging in the management of ureteral calculous disease: AUA technology assessment. J Urol.

[5] Rahmouni A, Bargoin R, Herment A, Bargoin N, Vasile N (1996). Color Doppler twinkling artifact in hyperechoic regions. Radiology.

[6] Bulakçı M, Tefik T, Akbulut F (2015). The use of non-contrast computed tomography and color Doppler ultrasound in the characterization of urinary stones-preliminary results. Turk J Urol.

[7] Lee JY, Kim SH, Cho JY, Han D (2001). Color and power Doppler twinkling artifacts from urinary stones: clinical observations and phantom studies. AJR Am J Roentgenol.

[8] Chelfouh N, Grenier N, Higueret D, Trillaud H, Levantal O, Pariente J, Ballanger P (1998). Characterization of urinary calculi: in vitro study of "twinkling artifact" revealed by color-flow sonography. AJR Am J Roentgenol.

[9] Kielar AZ, Shabana W, Vakili M, Rubin J (2012). Prospective evaluation of Doppler sonography to detect the twinkling artifact versus unenhanced computed tomography for identifying urinary tract calculi. J Ultrasound Med.

[10] Sorensen MD, Harper JD, Hsi RS (2013). B-mode ultrasound versus color Doppler twinkling artifact in detecting kidney stones. J Endourol.

[11] Ahmed F, Zafar AM, Khan N, Haider Z, Ather MH (2010). A paradigm shift in imaging for renal colic - is it time to say good bye to an old trusted friend?. Int J Surg.

[12] Rodger F, Roditi G, Aboumarzouk OM (2018). Diagnostic accuracy of low and ultra-low dose CT for identification of urinary tract stones: a systematic review. Urol Int.

[13] Niemann T, Kollmann T, Bongartz G (2008). Diagnostic performance of low-dose CT for the detection of urolithiasis: a meta-analysis. AJR Am J Roengenol.

[14] Curhan GC (2007). Epidemiology of stone disease. Urol Clin North Am.

[15] Dillman JR, Kappil M, Weadock WJ, Rubin JM, Platt JF, DiPietro MA, Bude RO (2011). Sonographic twinkling artifact for renal calculus detection: correlation with CT. Radiology.

[16] Masch WR, Cohan RH, Ellis JH, Dillman JR, Rubin JM, Davenport MS (2016). Clinical effectiveness of prospectively reported sonographic twinkling artifact for the diagnosis of renal calculus in patients without known urolithiasis. AJR Am J Roentgenol.

[17] Yavuz A, Ceken K, Alimoglu E, Kabaalioglu A (2015). The reliability of color doppler" twinkling" artifact for diagnosing millimetrical nephrolithiasis: comparison with B-Mode US and CT scanning results. J Med Ultrason.

[18] Gliga ML, Chirila CN, Podeanu DM, Imola T, Voicu SL, Gliga MG, Gliga PM (2017). Twinkle, twinkle little stone: an artifact improves the ultrasound performance!. Med Ultrason.

[19] Kamaya A, Tuthill T, M. Rubin J (2003). Twinkling artifact on color Doppler sonography: dependence on machine parameters and underlying cause. American Journal of Roentgenology.

[20] Hanafi MQ, Fakhrizadeh A, Jaafaezadeh E (2019). An investigation into the clinical accuracy of twinkling artifacts in patients with urolithiasis smaller than 5 mm in comparison with computed tomography scanning. J Family Med Prim Care.

